# Family Husbandry in the Tropical Island of Mayotte: Struggling for Autonomy from Production to Sanitary Problems

**DOI:** 10.3390/ani14233405

**Published:** 2024-11-26

**Authors:** Jacques Cabaret, Sittirati Mohamed, Fabrice Guégnard, Claude L. Charvet, Cédric Neveu, Mohamed Issouf

**Affiliations:** 1 SantéSocioVéto, 8 Place Carré de Busserolle, 37100 Tours, France; 2 MayBiotech SARL, 8 Rue de la Gendarmerie, 97620 Bouéni, France; sarl.maybiotech@gmail.com (S.M.); missouf976@gmail.com (M.I.); 3 Institut National de Recherche pour l’Agriculture, l’Alimentation et l’Environnement (INRAE), Université de Tours, ISP, 37380 Nouzilly, France; fabrice.guegnard@inrae.fr (F.G.); claude.charvet@inrae.fr (C.L.C.); cedric.neveu@inrae.fr (C.N.)

**Keywords:** husbandry, cattle, sheep, goat, chickens, autonomy, diseases

## Abstract

Mayotte is a small tropical island in the Comoros archipelago. It became recently a French department and much of its food, especially meat, is imported from abroad. The development of livestock farming is therefore a necessity. To understand the problems faced by Mahoran farmers, we organised semi-directive interviews with 15 farmers who reared cattle, sheep, goats or poultry. The first difficulty of farmers was limited access to land, especially for ruminants. This led to feed shortages. Another difficulty was the limited access to water and the poor quality of the roads to reach the farms. Poultry farmers were too dependent on importations of feed and laying hen or broiler genotypes from metropolitan France. The lack of organization for independent food productions (absence of abattoirs, cooperatives or organised markets) is also an obstacle to the development of the sector. Animal health, although not considered a major problem, has been a nuisance in the past (anthrax in cattle or salmonella in poultry). Mahoran farmers trust veterinarians or their assistants to manage health, although they complain about the high cost. Surprisingly, farmers use traditional medicine for many of their ailments, mostly based on local plants, but rarely for animals. Overall, our study reveals that larger land areas, better availability of money for investment and access to water and fodder are urgently required to improve livestock production and economic viability of farmers in Mayotte.

## Simple Summary

Family husbandry has its own characteristics in different regions. We used semi-directive interviews with fifteen farmers, who raise cattle, sheep and goats, or poultry in the tropical island of Mayotte, to identify their main concerns. The island’s history as a colony and more recently as a French department reveals that livestock farming faces a number of difficulties. The limited technical background of the farmers, the small size of the farms and the lack of marketing structures are among the main problems. Access to land is hampered by complicated and unregulated property rights. Public services in the island are not fully satisfactory, with limited access to water and poor-quality roads to farms. Animal health is not considered a major problem, and farmers rely on veterinarians or their assistants. Although Mahoran farmers use traditional medicine for themselves, they are reluctant to use it for their animals. Livestock production in Mayotte can be economically viable on larger farms with the capacity to invest.

## 1. Introduction

Mayotte is a French ‘département ‘(374 square kilometres) in the Comoros archipelago, located in the Indian Ocean between the eastern coast of Africa and Madagascar. The climate is tropical with average daily temperatures ranging from 26 to 28 °C along the year; the total annual rainfall amounts to 1200 mm and the dry season extends from June to October. The Mahoran density of population is high (690/km^2^), making the food and water supplies a critical issue.

Due to a limited production, much of the food and particularly meat is imported frozen from metropolitan France or other countries. The farms are of small size and the island’s livestock production includes mostly cattle (21,000), goats (11,500) and poultry (225,000; mostly broilers and egg laying hens) [1]. Agriculture subsidies, linked to farm size, represent 10% of direct help to the farmer’s income, with most of the farms having a very small area (1.5 ha/farm) [1,2]. The characteristics of the cattle production have been described as professional (more than 12 animals, practice of artificial insemination, and use of supplementary external feed, with a tendency to increase the size of their herd) and traditional (use only of local food with no attempt to increase the size of herds) [3]. The absence of slaughterhouses is a problem in maintaining adequate sanitary surveillance [4] and the local production is mostly absorbed in festive occasions (marriages, and Muslim feasts). A participative epidemiological survey was performed previously [5], revealing anthrax, ticks, a viral disease (probably blue tongue), dermatophilosis, diarrhoea and internal parasites as the most frequent diseases of cattle considered by farmers. The main difficulties in dairy farms include the provision of feed and water, but high mortalities and damages are also caused by stray dogs [6]. The production of broilers [7] and eggs [8] has been evaluated from an economical point of view, and the difficulties related to import small chicks as well as the organisation of production and marketing have been pointed out. The knowledge on husbandry in Mayotte is mostly based on expert reports and the views of farmers remain largely unknown. In that respect, our survey intended to outline their opinions on husbandry (cattle, goats and chickens) and their practices, particularly on animal health. Here, we will estimate the level of autonomy of farmers from their interviews. Autonomy is the ability of farmers to decide on all aspects of farm management (from feed to production objectives and good health maintenance) as opposed to integration by big firms or the state. It is very similar to local integration at the farm level when the farmer decides on the complementarity of agriculture and husbandry, which animals to be bred, the food management, housing, therapeutics [9] and commercialisation of production, as it was described in meat sheep farms of France and Algeria [10]. However, these decisions may be limited in Mayotte when the availability of land is scarce and land tenure system is complicated [11], with poor quality of roads to the farms and access to water to only a third of households [12]. Hence, we also investigated the importance on autonomy of these limiting factors among farmers.

## 2. Material and Methods

### 2.1. Farms

**Figure 1:**
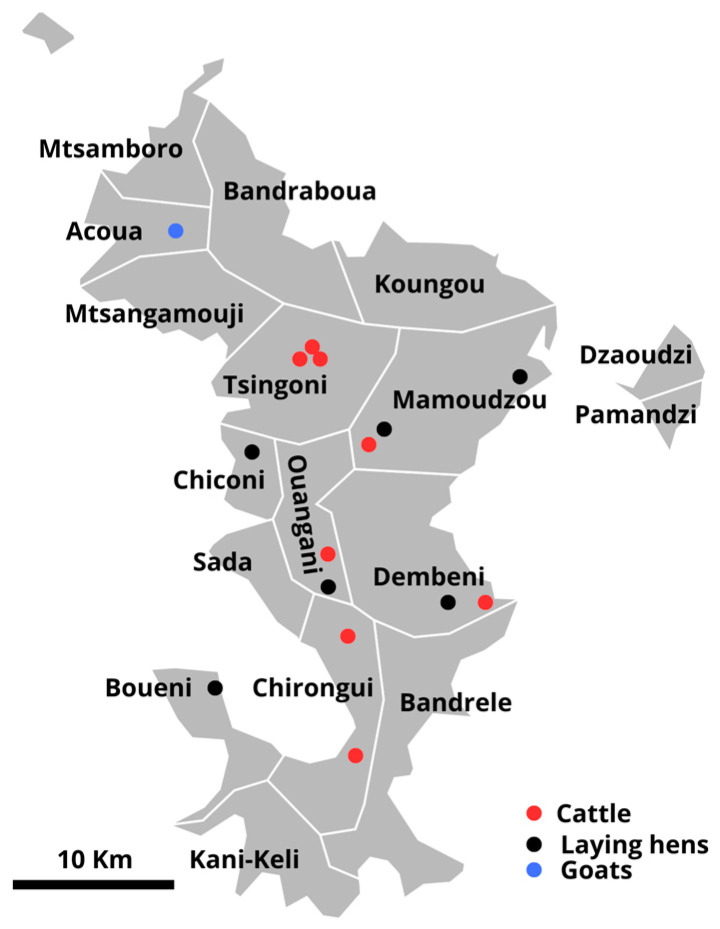
Location of the studied farms in Mayotte.

The farms were selected by one of us (SM) on a list of the former farmers’ cooperative of Mayotte (Coopérative Agricole Des Eleveurs Mahorais-COOP ADEM) and on their willingness to participate in the survey. They were breeding cattle, sheep and goats, or chickens or ducks and were all located on the island of Grande-Terre of Mayotte (Figure 1).

They were family farms (Supplementary Figure S1a,b), although they were considered as professional or intending to become professional as described by [3]. They were considered good farms because the owners were former members of the cooperative and have been offered technical services.

### 2.2. Interviews

A visit of the farm was carried out before the interviews that lasted approximately half an hour, either in French or in Shimaore, a local version of Swahili (4 out of 15 farms) that was translated by one of us (M.I.). The interviewers (J.C., S.M. and M.I.) asked open/semi-directive questions to farmers as described by [13]. The questions had been pre-prepared in an interview guide, which was similar to the one used in a previous study in French Western Indies [14]. After testing the interview guide in the first farms, it was clear that it should be simplified and most of the questions remained fully open (Supplementary Data S1). Briefly, the questions were asked about their personal and professional backgrounds, farm management and work, participation in collective activities, animal health and treatments used, how they gained experience in animal health and, finally, how they saw the future of their profession. It was identical for all interviews. In addition, an interview of a veterinarian dedicated in part to farm animals was performed to measure the presence of veterinarians and veterinary assistants in these small farms. The interviews were anonymized.

### 2.3. Analyses of Interviews

The recorded interviews were transcribed into a Word text. Tropes (V8.5) [15] speech analysis software was first used to process the data on cognitive analysis of the interviews [16] and then analysed using multivariate methods [17] applied to the most frequently used words in the interview. The difference between discourse analysis in our study and textual linguistics is that discourse analysis aims to reveal the socio-psychological characteristics of a farmer rather than this textual structure. We wanted to identify homogeneous groups of farmers, as has been carried out previously with horse breeders [18]. Significant differences between homogeneous groups of stakeholders were assessed using Z score statistics for two populations; where the proportions were low (less than 4%), Fisher’s exact test was applied to the number of occurrences for each word. The classical way to interpret interviews was also used: the most exemplary sentences were selected by the interviewers and combined to describe the management and problems in husbandry.

## 3. Results

### 3.1. Farms’ Diversity

The characteristics of the farms are presented in Table 1. They were all small family farms, except for F4, which was larger and had diversified livestock production and integrated marketing. The main production was cattle for milk and meat and laying hens for eggs. The age of the farmers ranged from 30 to 70 years and they had all been farming for at least 10 years. Only four out of fifteen were less than 40 years old. Three farms were run by women, one by a couple, and the others by men. Two farmers had work other than husbandry, such as being an employee elsewhere. One sold her products in her restaurant and butcher shop. All of them had another production-like market garden or fruits or own garden. Only one had formal training in animal husbandry, while the others learned on the spot with their family or in short sessions organised by various stakeholders. Ten out of fourteen had never used complementary or alternative medicines on their animals.

### 3.2. The World of Farmers as Described by Word Occurrences

We recorded the percentage of each word mentioned during the interviews. The most frequently mentioned health advisors to farmers were veterinarians (26%) and agricultural technicians (7%). Treatments accounted for 7% of cases and parasites 4%. The use of plants in treatments was very limited (3%). Agriculture (13%) was mentioned as most farms had both livestock and agriculture. The main problems in animal husbandry were lack of access to water (12%) to feed the animals on the farm (7%), scarcity of available land (5%), theft of animals (4%) and poor quality of roads (3%, Supplementary Figure S1c) to transport food and materials to the animals. This made the work more difficult (5%) and therefore other financial or technical help was needed from the state (6%). It may also explain why the word children was cited (6%) to indicate the lack of interest for husbandry among the sons and daughters of these farmers.

There were several differences between cattle and avian husbandry (Table 2). The cattle farmers insisted more on the role of veterinarians than the avian farmers, who were given a standard vaccination and treatment protocol to be followed. Feeding the animals was more of a problem for avian farmers since they are dependent on the importations from metropolitan France. That is why they probably insisted on the need of the state for managing importations of feed and young chickens. Both farmers selected veterinarians and access to water as major roles in their farms. Feed importation and rainfalls highly varied according to the year.

### 3.3. Multivariate Analyses of Farmers’ Problems

**Figure 2:**
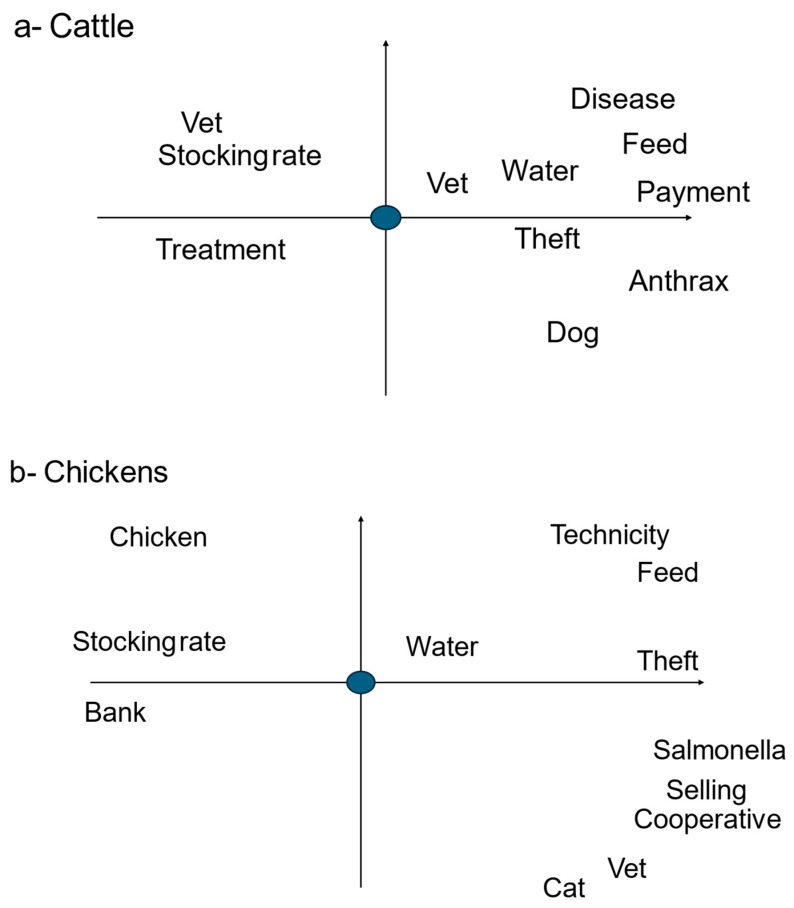
Multivariate analysis of farmers interviews with Tropes in cattle and chicken farms in Mayotte (Tropical France): relation of the word problem (blue dot) with characteristics of their environment. The left part of the graph corresponds to the beginning and the right part to the ongoing interview. The words are exactly located on the figure at the place of their first letter. Words in the same location on the figure are related. The words near the black dot are strongly related to the word ‘problem’ and those far away are only loosely related.

The multivariate analysis of interviews with Tropes on the problems they face, and their environment is presented in Figure 2. Cattle farmers (Figure 2a) see the vet (or their assistant) as the person to contact for treatment and diseases. High stocking rates, availability of water, animal theft and stray dogs are seen as major problems for farmers, more so than diseases (including anthrax). Other problems are animal feeding and lack of cash. Chicken farmers (Figure 2b) also have a major problem with the availability of water. The veterinarian appears to be less important than in cattle farming, as they rely on the veterinarian to provide standard protocols for maintaining health, which remain unchanged. The problems are many: difficulties in increasing the size of the farm (stocking rate—linked to difficulties in obtaining bank loans), difficulties in obtaining young chickens to breed, the lack of a cooperative to improve technical knowledge and the sale of products, feeding the animals as it depends on the flow of imports, theft and robbery by stray cats, and finally the fear of salmonella infection, which prevents any sale.

### 3.4. Farmers’ Constraints in Their Own Words

#### 3.4.1. Reliance on Metropolitan France

Much of the feed originates from metropolitan France: “The problem is to obtain feed from metropolitan France” (F13); “I have had a big crisis in 2018: we could not get imported feed because of strikes… The feed is prepared from imported products (maize…)” (F14). The cattle are often imported from metropolitan France: “I tried Brown Swiss, Limousin and then Montbéliarde breeds. I had plenty of problems, foot disease, dermatophilosis. It was catastrophic with the Alp brown” (F2); “We inseminate with Brown Swiss or Montbéliarde, we do not take Holstein” (F7), or African zebu. The chicks originate from metropolitan France: “We get Isa brown… It is not always easy to get them, because a direct flight is needed for their transport, which is not frequent during summer holidays” (F14).

#### 3.4.2. Deficient Public Services, Land Tenure System and Security

Running water is not available in many farms, and it remains a main constraint either for cattle (“The problem is water”—F12) or chicken husbandry: “There is a lack of water during the dry season. It comes by tanker truck, but we have asked for establishment of running water…” (F6); “There is a problem with water… I will install a well” (F15). Electricity is not also available in several farms (F15). One other major complaint is related to the absence or bad quality of the road to access the farm: “We don’t have a road to the farm, we need one” (F9); “The road to access to the farm is really bad” (F8). The quality of public service may be a cornerstone for the transmission of the farm: “If there is water, electricity, a road, our children will follow up” (F9). Lack of available land is mentioned in the following statements: “I breed goats because I do not have enough plots, I do not have enough space…The land belongs to the olds” (F5); “I have a problem with land use since I am in a forest zone. I do not have any justification to use them. I have two sites” (F10). The security of animals is not satisfying since thefts are frequent: “I have a problem of thefts of animals, and I hire a watchman” (F9) and “The best is to have a hut and watch the animals” (F15). There have also been issues with stray dogs (“I have had stray dogs frequently and I solved the problem with fencing around the animals” (F4)) and cats (F9).

#### 3.4.3. Access to Technical Knowledge and Improved Animal Health

Since many farmers are not trained initially in husbandry, they are requesting training and help from technicians in the domain with different levels of satisfaction: “I did not get any training for the last two years” (F4); “I obtained training with the cooperative, the Chambre of agriculture” (F13); “I trained with meeting, seminars, for example on agrobiology in Madagascar” (F2). They largely use chemicals or vaccinations as recommended by the veterinarian or his assistant: “If I have a calf with Ascaris, I go to fetch the product at the veterinarian and I treat it” (F2); “One single disease can destroy a farm. The chicks are vaccinated, and we follow the programme given by the veterinarian” (F11). They however complain of the cost of the veterinarians: “I treat myself the simple things like abscesses… To ask the veterinarian costs you a heap of money” (F7). The available health structure is not very stable since one veterinarian with a veterinary assistant are supposed to monitor up to 540 farms (the vet interviewed). The use of traditional medicine for animals (mostly cattle) remains limited although it is a frequent occurrence for humans: “I believe in traditional medicine for myself, but we have to be careful when using it for animals” (F12); “I give salted water when I do not have money to pay the veterinarian” (F13); “We water the backs of cattle with tea of several plants as taught by my father. I lost much of practice since we did not write the recipes” (F7). Few recipes are used, including salted or sea water in four farms, *Aloe vera* and wild grapefruits (F1), eucalyptus, lemon water, lemon grass (F7) and nettle juice for cow infertility (F10).

## 4. Discussion

The autonomy of family husbandry depends on the general environment, the administrative conditions and the technical knowledge of the farmers [10]. They have no control over agricultural land availability, water and fodder supply, sanitary conditions and the possibility of marketing the products. During the colonial period in Mayotte (1911 to 2008), there was a typical land tenure system close to that of Madagascar [11]. This system came from the Australian Torrens Act system, which was based on registration, a long and cumbersome process that issued a title deed to the farmer. This title was perpetual and inviolable, but it was also optional. From the creation of the cadastre in 1993 to the land taxation in 2014, the alignment with the French law is still problematic due to inertia (of owners and administration) and the fact that a significant proportion of the population of Mayotte simply occupy land and do not own it [19] (see F10). As a result, farms are too small and cannot be expanded. The profession is not very attractive due to the low income and the workload combined with the lack of possibility to enlarge and modify [1,3] the farm. Limited access to land is one of the major challenges in scaling up production and addressing local food demand. In addition, water supplies and road connections are not in the hands of the farmers, which further reduces the attractiveness of the profession. Therefore, the average age of farmers is high and most of them do not expect their children to follow them. However, some younger farmers (see F14 and F15) with agricultural education (mostly for chickens) are undertaking farming and may give hope for animal production in Mayotte.

Globalisation and post-colonisation are interconnected [20] and influence the local autonomies. A post-colonial situation [21] reduces autonomy: cattle breeds are imported (Montbéliarde, Brown Swiss, Limousin) to the detriment of local zebus, and day-old chickens (and sometimes their feed) are all imported from metropolitan France. Moreover, globalisation has fostered the importations of frozen beef and chicken meat, and subsequently have changed eating habits, which explains, for example, the popularity of chicken wings (‘mabawa’ in Shimaore) [22]. Most local farmers cannot provide the meat in high quantities and at a cheap price that is consumed by most of the population on a daily basis. The local farmers then offer animal products mainly for festive occasions. Nevertheless, larger farms providing fresh milk and meat do not have problems selling their products on a regular basis as seen in F7, since a part of the population is fed up with frozen meat from abroad [22]. The neocolonial situation also exists in other tropical islands, such as the French West Indies, but to a lesser extent [23,24]: local Creole breeds are available, farm sizes are larger, the land tenure system is similar to that in metropolitan France and technical knowledge is developed, but they also face unfair competition from imported agricultural products.

Farmers rely on vets or their technicians for health management. This is a common situation in Greece [25], the French West Indies [14] and South Africa [26], among other countries. In the latter study, the determinants of the farmer’s choice of primary animal health care practices showed that access to animal handling facilities, contact with a vet, membership of a farmers’ association, household income and positive perceptions of vaccines had a positive influence on the farmer’s choice of these health care practices. Similarly, farmers in Mayotte were demanding of structures, associations and veterinarians or their assistants. They were also limited by their income and some had doubts about vaccination (against anthrax) [5]. The autonomy of Mayotte’s farmers is limited to the detection of sick animals, but they rely on vets to suggest medicines to deal with health problems. This is somewhat surprising because traditional medicine is frequently used on humans [27], either on a family basis or by traditional healers (‘foundi’ in Shimaore). There are even syndromes that do not correspond to the Western description, such as ‘muamusi’, which corresponds to a pain in the lower abdomen with many different symptoms [28]. Although the ‘foundis’ propose cures for 65 human diseases, from hypertension to diabetes [29], very few local veterinary treatments are frequently used (sea water, aloe) and they are identical to those practised in the French West Indies [14]. The situation is different in East Africa, where traditional medicine combined with plants is used to treat both humans and domestic animals [30]. Traditional medicine is the only resource for poor people, but the people of Mayotte have a higher economic capacity and knowledge of traditional animal medicine has nearly disappeared (F7). Farmers admit that they are afraid to use traditional animal medicine due to their lack of knowledge, and their use of complementary/alternative medicine based on plants needs to be supported by experimental demonstrations.

## 5. Conclusions

The autonomy of farmers on the island of Mayotte is severely limited by their lack of technical knowledge, low incomes and restrictions on land acquisition. There is also a need for better roads, water supply and an organised market to encourage the development of livestock farming. If all these constraints are reduced, there are opportunities for economically sustainable farming in Mayotte. The development of scientifically based complementary/alternative medicine could also be of interest, as many plants are available locally to treat various human ailments.

## Figures and Tables

**Table 1:** Characteristics of the farms of Mayotte.

Farms	Main Production (Number of Animals)	Secondary Production	Age	Gender (Female F, Male M)	Husbandry Knowledge	Use of Alternative/Complementary Drugs
F1 *	Cattle (10), meat.	Goats (8), market garden	70	F	Practical	Sodium bicarbonate, sea water
F2	Cattle (30), meat.	Fruits	60	M	Practical	None
F3	Cattle (9), milk and meat.	Market garden	40	M	Practical	None
F4	Cattle (50), meat and milk.	Broilers (100), laying hens, restaurant, butcher’s shop.	35	F	Practical	*Aloe vera*, salted water, eucalyptus, lemon grass.
F5	Cattle (13), meat.	Goats (20), employed in other work.	40	M	Practical.	None.
F6	Cattle (22), meat.	Goats (30)	70	M	Practical.	*Aloe vera*, sea water.
F7 *	Cattle (8), milk.	Goats (10)	55	M	Practical.	Several juices of plants.
F8 *	Cattle (11), milk.	Ducks (30)	50	M	Practical.	Salted water.
F9	Goats (50), meat.	Employed in other work.	40	M	Practical.	None.
F10	Laying hens (3000).	Market garden.	40	F	Formal agricultural education.	None.
F11 *	Laying hens (300).	Ducks (300), production of coffee	50	F, M	Practical.	None.
F12	Laying hens (2000).	None.	35	M	Formal agricultural education.	None.
F13	Laying hens (6000).	None.	50	M	Practical.	None.
F14	Laying hens (900).	Fruits.	30	M	Practical.	None.
F15	Laying hens (3000).	Cattle (3), fruits.	30	M	Practical.	None.

* Interviews in Shimaore; others in French.

**Table 2:** Words used by cattle and avian farmers and their frequency during the interviews.

Domain	Words	Occurrence (%)		Significance
		Cattle Husbandry	Avian Husbandry	
Health	Veterinarian	29	18	<0.10
	Treatment	7	7	ns
	Vaccination	1	5	ns
	Technician	1	5	ns
Husbandry	Feeding	2	11	<0.05
	Access to water	17	10	ns
	Husbandry buildings	2	5	ns
Learning	Technical education	7	3	ns
Economy	Euro	2	7	ns
	Help from the state	3	12	<0.05
Temporality	Year	15	12	ns
	Season	5	0	ns
	Months	9	5	ns

## Data Availability

The original contributions presented in the study are included in the article/supplementary material, further inquiries can be directed to the corresponding author.

## Supplementary Materials

The following supporting information can be downloaded at https://www.mdpi.com/article/10.3390/ani14233405/s1: Data S1: Guide for interviews on autonomy and the health experiences of Mayotte farmers. Figure S1: a,b,c: Family farms (chickens, goats and a road to a farm).
