# Supplementary material for: Family Husbandry in the Tropical Island of Mayotte: Struggling for Autonomy from Production to Sanitary Problems
Source: Animals (Basel). 2024 Nov 26;14(23):3405. doi: 10.3390/ani14233405 (PMC11639882; doi:10.3390/ani14233405)
Supplement: Supplementary file 1 [file animals-14-03405-s001.zip › Farmers'interviews' guide.pdf]

Farmers' animal health experiences and their use of alternative therapies.

Before each interview, which will be recorded with the breeder's agreement, we will inform him/her that the data will be anonymised and used for research purposes.

1- Personal and professional background

History of the breeder and his profession

How did you learn the trade?

How has your farm evolved?

2- The husbandry and work on the farm

Is livestock farming your main activity?

Current technical characteristics of the farm: how many people work on the farm, number of animals, which species and why, surface area, feed autonomy,

Are there several jobs in husbandry: which ones?

3- What collective activities?

Technical exchanges within the farm and outside the farm: neighbours, technicians, vets, etc...

With whom do you discuss your work, are you in technical discussion groups?

4- How do you evaluate work in husbandry?

What is good husbandry?

What are beautiful animals?

5- What is the animal health situation on your farm?

What is the past health history on your farm?

What are the first indicators of health problems?

Who did you rely on to solve them? Colleagues, technicians (which ones), vet...

What is a normal health situation in your farm?

6- Health treatments

Synthetic allopathic treatments?

What plant products do you use?

Why did you choose them?

Other treatments?

7- How did you build up your health experience, on what occasions, with whose help?

Which other farmers helped you and how?

Which technicians?

Which veterinarians?

How do animals contribute to their care by self-medicating?

8- How are farming methods and the profession changing?
