# Supplementary figures and images for: Family Husbandry in the Tropical Island of Mayotte: Struggling for Autonomy from Production to Sanitary Problems
Source: Animals (Basel). 2024 Nov 26;14(23):3405. doi: 10.3390/ani14233405 (PMC11639882; doi:10.3390/ani14233405)

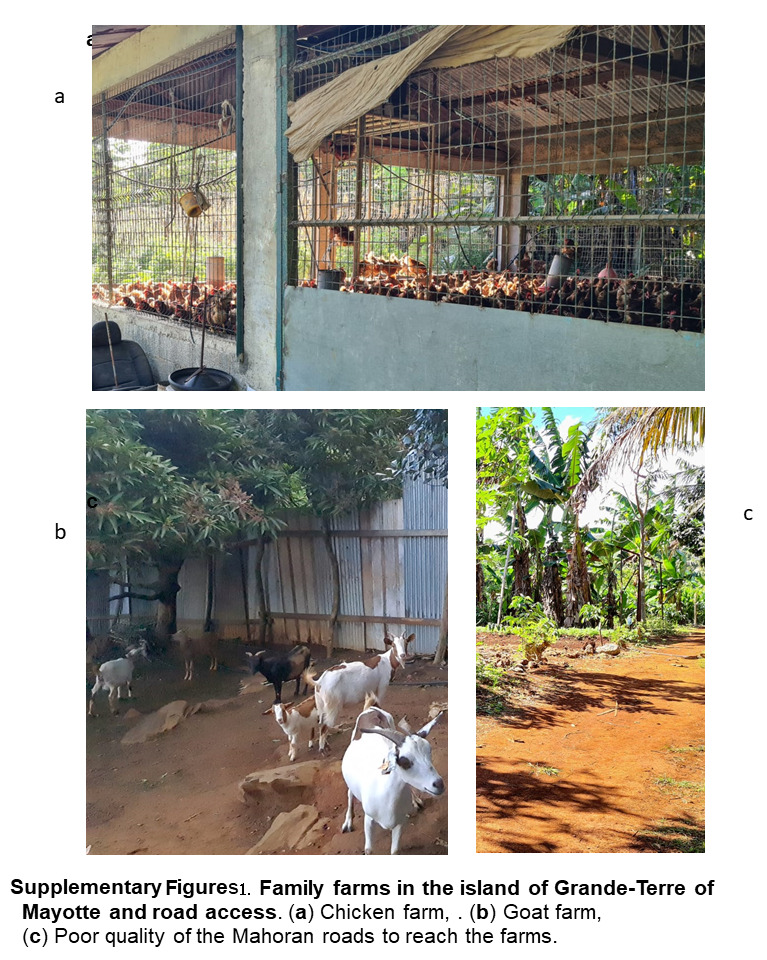

Supplement: Supplementary file 1 [file animals-14-03405-s001.zip › Supplementaryfigure a,b,c.png]
